# Multimodality imaging and advanced calcium treatment to facilitate PCI in a rare coronary artery anomaly—case report

**DOI:** 10.3389/fcvm.2025.1471211

**Published:** 2025-05-21

**Authors:** Kosta S. Krupnikovic, Danilo Obradovic, Ivan Ilic, Milan Dobric

**Affiliations:** ^1^ Institute for Cardiovascular Diseases ‘Dedinje’, University of Belgrade, Belgrade, Serbia; ^2^ Department of Internal Medicine and Cardiology, Herzzentrum Dresden, Technische Universität Dresden, Dresden, Germany; ^3^ Faculty of Medicine, University of Belgrade, Belgrade, Serbia

**Keywords:** coronary artery anomaly, intravascular ultrasound, intravascular lithotripsy, PCI, CTCA

## Abstract

**Background:**

Coronary artery anomalies (CAAs) are a rare congenital condition and represent additional challenges in interventional treatment of coronary artery disease.

**Case summary:**

A 76-year-old male, was admitted for elective coronary angiography due to symptoms of typical angina. CT coronary angiography (CTCA) revealed all three coronary arteries arising from the right sinus of Valsalva, where right coronary artery (RCA) and left anterior descending artery (LAD) had common ostium with significant stenosis of ostio-proximal RCA and circumflex artery (CX) coming from a separate one. Percutaneous coronary intervention (PCI) of ostial RCA was planned and intravascular ultrasound (IVUS) in both RCA and LAD was done. Due to extensive calcification, prior to intended PCI, intravascular lithotripsy (IVL) was done. Following IVL and extensive predilatation drug eluting stent (DES) was implanted. Final IVUS was used to confirm optimal stent deployment in proximal RCA and to verify that LAD ostium was not compromised with RCA stent. Six months later, due to angina and positive stress test, repeated coronary angiography revealed a restenosis of the ostial RCA so the lesion was again treated with drug-coated balloon with optimal procedural results.

**Conclusion:**

Although rare, CAAs could be associated with coronary artery disease and usually present additional challenge for interventional treatment. Advanced imaging modalities, including CTCA and IVUS, provide good procedural guidance during complex PCI procedures in patients with CAAs.

## Introduction

Coronary artery anomalies (CCAs) are a rare congenital condition and represent additional challenge in interventional treatment of the coronary artery disease (1). In the systematic review of 12.457 consecutive adult patients who underwent coronary angiography, origin of all three major coronary arteries at right coronary sinus was found to have a prevalence of 0.008% and comprised 0.89% of all congenital anomalies found in the study population (2). The intravascular ultrasound (IVUS) has already been proven to improve outcomes in complex coronary interventions (3). Compared to coronary angiography guided PCI, the use of IVUS imaging guidance to optimize stent implantation was associated with a lower risk for target vessel failure (TVF) during long term follow-up (4). The benefits of intravascular imaging have been especially pronounced in treatment of calcified lesions which comprise substantial part of contemporary PCI (5). IVUS definitions of calcium burden allowed evaluation of new techniques to treat calcified lesions like atherectomy or intravascular lithotripsy (IVL) (6). In the Disrupt CAD III trial, the largest prospective single-arm multicenter study to date, IVL treatment prior to coronary stent implantation in severely calcified lesions was associated with lower rates of major adverse cardiac events, ischemia-driven target lesion revascularization and stent thrombosis at one-year follow-up (7).

## Case report

**Figure 1:**
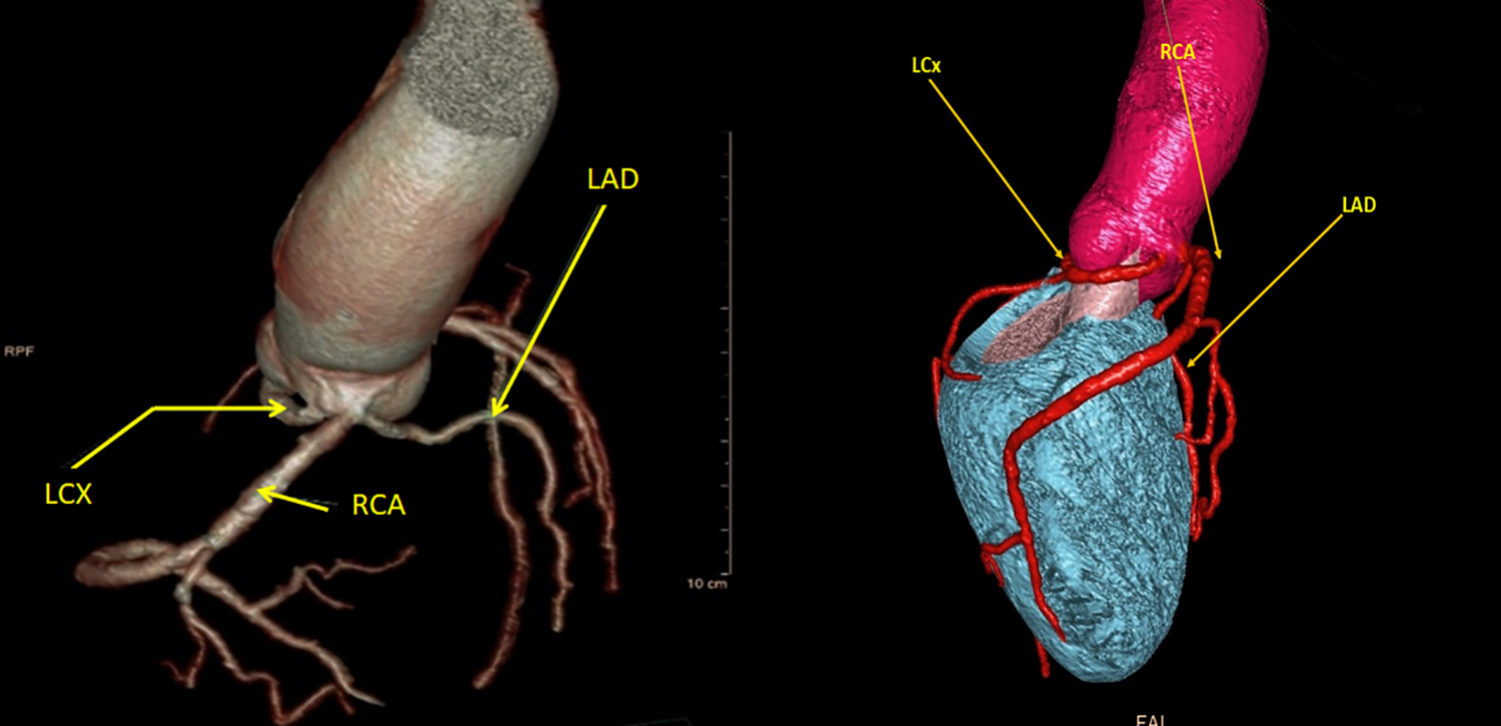
Multi-slice computed tomography (MSCT) of the coronary arteries. All three coronary arteries emerge from right sinus of Valsalva. RCA and LAD emerge from same ostium and lay in close proximity. LCX is positioned laterally and emerges from isolated ostia.

A 76-year-old male patient, with no relevant medical history, was admitted for coronary angiography due to symptoms of typical angina. Patient underwent coronary angiography which was unable to provide enough information regarding coronary anatomy due to inability to selectively cannulate all coronary ostia. Afterwards, CT coronary angiography (CTCA) was done, which revealed the origin of all three coronary arteries from the right sinus of Valsalva. Right coronary artery (RCA) and left anterior descending artery (LAD) had common ostium and circumflex artery (CX) had separate ostium of its own and retro-aortic course. It also showed a high level of calcification in the ostioproximal segment of the RCA, which was later confirmed using intravascular imaging which demonstrated a high level of calcification occupying more than 270° of vessel circumference (Figure 1).

At repeated coronary angiography, two high grade stenoses were found in RCA, in ostioproximal and distal segment (Supplementary Videos 1, 2). The procedure was continued with PCI of the distal lesion first via radial access. The cannulation of RCA/LAD ostia was obtained using right Amplatz 2.0 7Fr catheter, which provided good support. Afterwards, two workhorse wires, *Whisper MS and BMW Universal* (Abbott Vascular International, Diegem, Belgium) were placed in distal segments of RCA and LAD, respectively. The predilatation of the distal RCA lesion was done with semi-compliant balloon 2.0 mm × 15 mm. After that, an everolimus eluting stent 2.5 mm × 20 mm was implanted in distal segment of the RCA.

**Figure 2:**
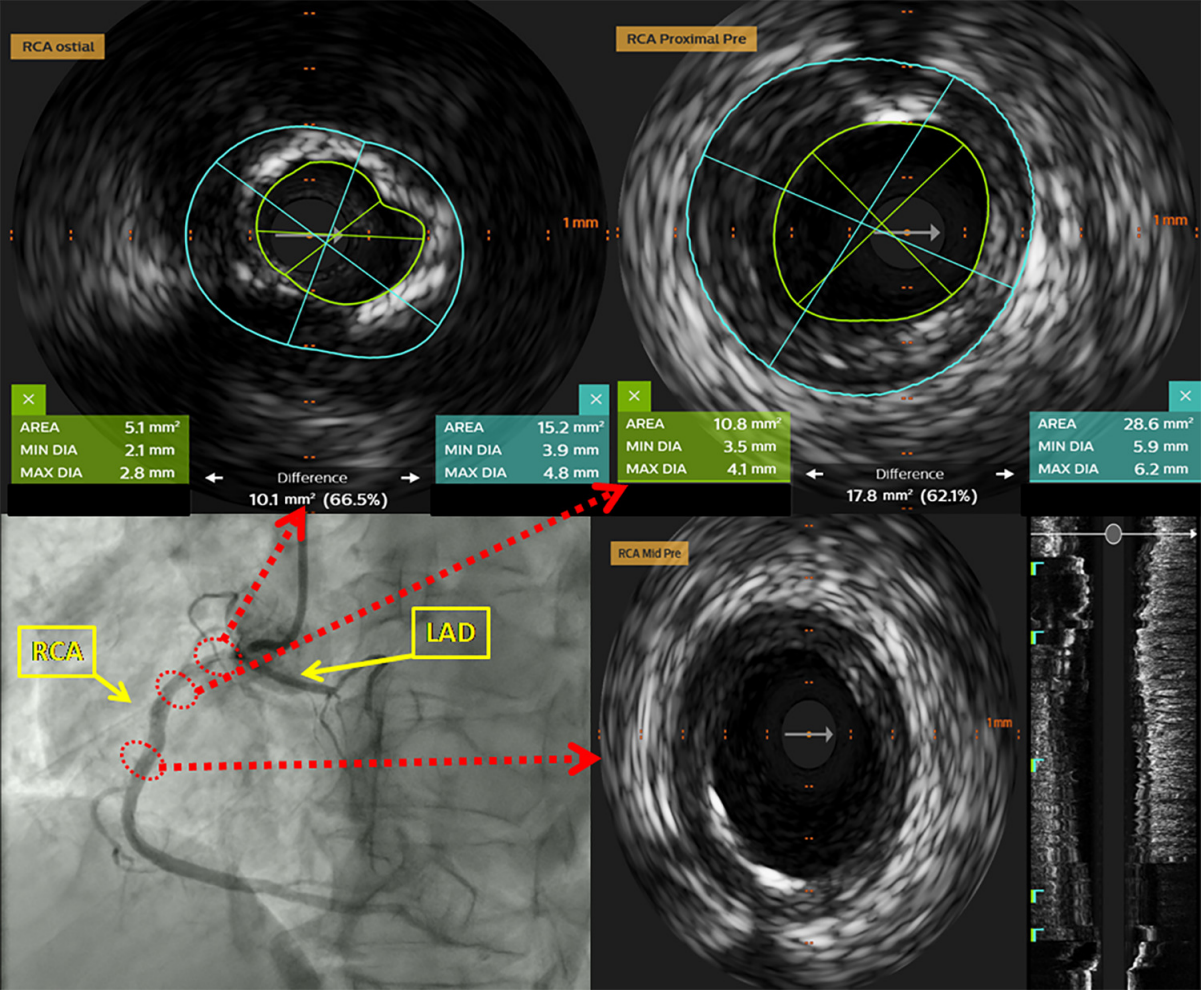
Intravascular ultrasound (IVUS) of the RCA prior to PCI. At the level of the ostial RCA segment highly calcified lesion was identified (more than 270° of vessel circumference). In the proximal segment of the RCA predominantly fibromuscular lesion was documented.

**Figure 3:**
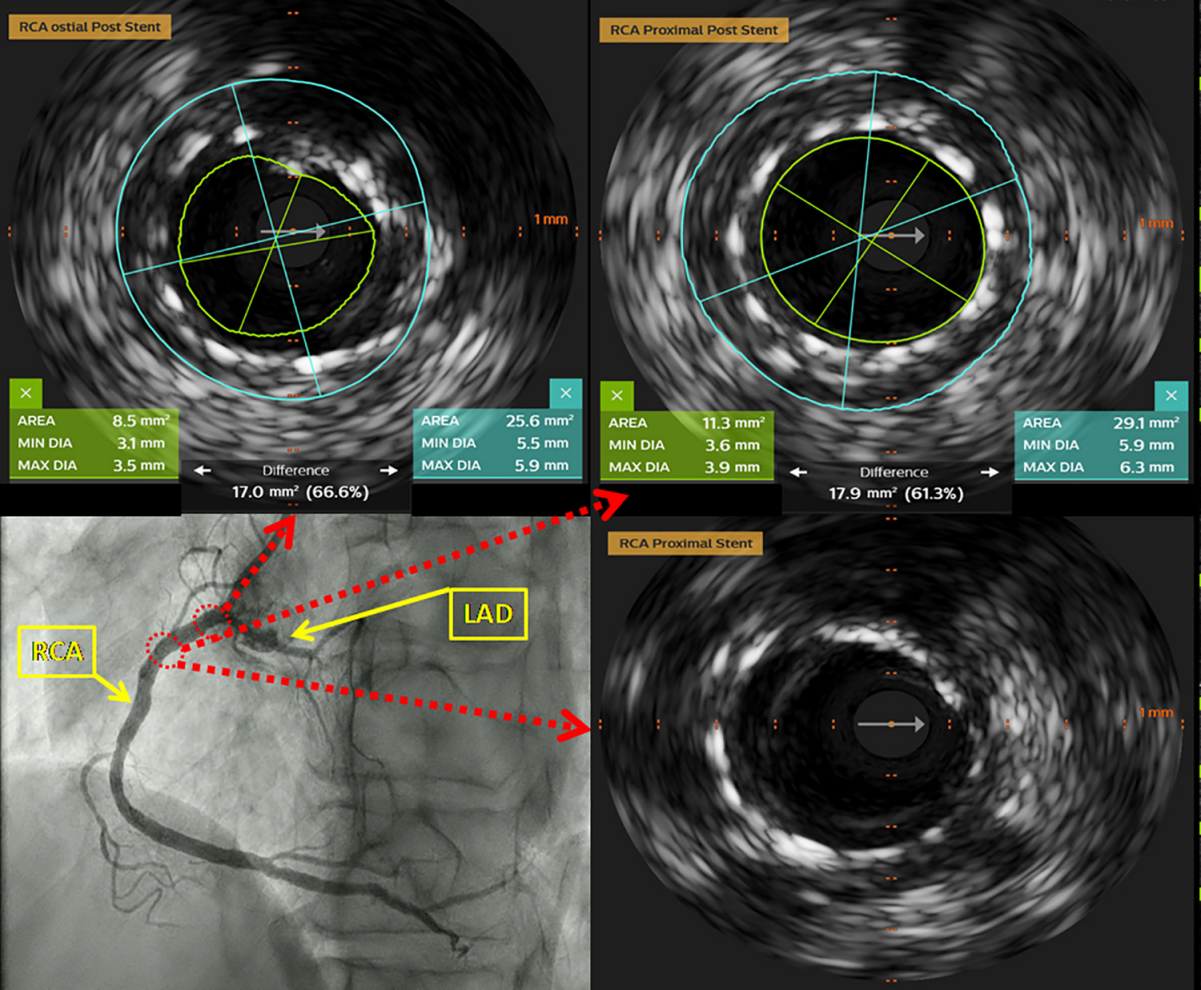
Intravascular ultrasound (IVUS) of the RCA after PCI. The optimal stent apposition without signs of dissection on proximal and distal stent edge was observed.

**Figure 4:**
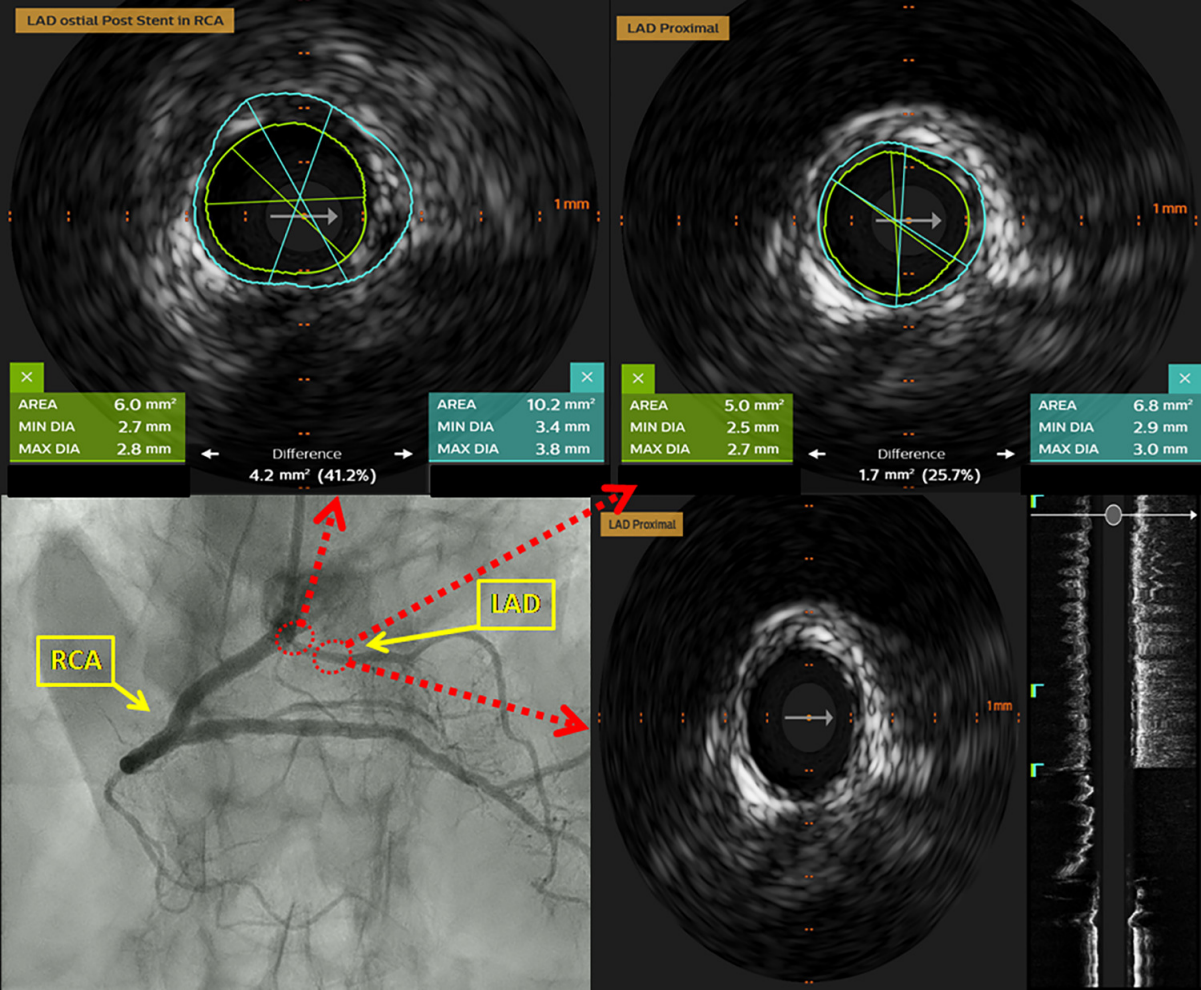
Intravascular ultrasound (IVUS) of the LAD after PCI. No signs of the narrowing of the ostial and proximal LAD after PCI of proximal RCA was documented.

Due to proximity of LAD to diseased RCA ostia, IVUS of RCA and LAD was done using Eagle Eye Platinum IVUS catheter (Philips Healthcare Andover, MA, US) (Supplementary Videos 4 and 5). At RCA ostium, stenosis was documented [minimal lumen diameter: 2.1 mm; maximal lumen diameter: 2.8 mm; lumen area (LA): 5.1 mm^2^; external elastic lamina (EEL) area: 15.2 mm^2^, plaque burden 66.5%] with extensive calcified plaque occupying more than 270° of vessel circumference. In the proximal segment predominantly fibro-muscular lesion (minimal lumen diameter: 3.5 mm; maximal lumen diameter: 4.1 mm; LA: 10.8 mm^2^; EEL area: 28.6 mm^2^, plaque burden 62.0%) was identified (Figure 2). No significant narrowing of LAD was observed. Due to extensive calcification of RCA ostium, we considered rotational atherectomy and IVL for lesion preparation. We opted for the latter one, owing to the challenges and higher risk of complications with rotational atherectomy given the proximity of the lesion to the ostium. After predilatation with non-compliant balloon 3.0 mm × 8 mm, IVL-balloon catheter 4.0 mm × 12 mm was positioned in the target lesion and inflated to 4 Atm (Supplementary Video 6). In total 9 cycles of IVL pulses were applied (Shockwave Medical, Inc, Santa Clara, CA). Following that, everolimus eluting stent Resolute Onyx 4.0 mm × 22 mm (Medtronic Inc, Galway, Ireland), was implanted in the proximal segment of RCA. Final IVUS runs were done from RCA and LAD and confirmed optimal stent deployment in RCA (minimal stent area at ostium: 8.5 mm^2^; minimal stent area at proximal segment: 11.3 mm^2^). There were no signs neither of dissection nor compromise of LAD ostium (Figures 3, 4, Supplementary Videos 7–10).

The patient was discharged on the following day on dual antiplatelet therapy including aspirin and clopidogrel. At one month follow-up visit, the patient reported doing well without angina.

However, at the next follow-up visit, six months after the initial procedure the patient reported having similar angina-like symptoms as prior to the PCI. Exercise stress test was done which showed signs of myocardial ischemia at levels of effort of 7.0 METS, with Duke treadmill score of −22 so repeated coronary angiography was done.

It revealed a significant restenosis of the ostial RCA, so the procedure was continued with repeated PCI (Supplementary Video 11). This time a Multi-Purpose 6F catheter was used, with two workhorse guidewires placed into the distal parts of the RCA and LAD. After predilatation with non-compliant and cutting ballon, the lesion was treated with a paclitaxel-coated 4.0 mm × 15 mm balloon for 60 s (Supplementary Video 12). The result was optimal and the patient was discharged from the hospital on the next day, with continuation of previously used dual-antiplatelet therapy (Supplementary Video 13).

## Discussion

The widespread use of invasive and noninvasive coronary artery imaging has led to increased recognition of CAAs among adults (8). Although the data concerning impact of CCAs on clinical outcome of patients are limited, there is evidence that specific morphologies of CCAs are associated with unfavorable clinical outcomes (9, 10).

CTCA offers a detailed characterization of the anatomic features associated with high-risk CAAs. Furthermore it allows visualization of the surrounding cardiac and non-cardiac structures and their three dimensional relationships, thus representing the gold standard for CAAs (11). In our case CTCA proved to be useful in providing detailed information regarding coronary artery morphology, their relationships, and the degree of coronary atherosclerosis (12).

Due to the complex anatomy present, the myocardial territory supplied by RCA and LAD is similar to the territory of the left main trunk. Therefore, the planned PCI should be performed with utmost caution, in order to minimize the risk of complications that would jeopardize large myocardial area and ensure better clinical outcome. Due to the same reason we have considered RCA ostial lesion as significant based on IVUS findings and did not pursue functional evaluation of the lesion.

Early IVUS studies showed that an angiographically good stent result frequently conceals poorly expanded struts with edge dissections that inevitably increases the risk of acute complications. Consecutive randomized trials proved, that IVUS-guided PCI provides better results than angiography guided PCI (13). Comprehensive information about the nature of the coronary plaque (calcified, fibromuscular, etc.), provided by IVUS, enables appropriate procedural decision making regarding lesion morphology and interventional strategies (14). Additionally, application of IVUS defined criteria for optimal stent implantation (plaque burden at 5 mm proximal or distal to stent edge <50%, minimal in-stent lumen area >90% of the distal reference lumen and absence of large edge dissection) provided in landmark clinical trials were associated with lower rates of MACE at one year follow-up (15, 16). Notwithstanding, optical coherence tomography (OCT) could be an alternative for plaque and lesion evaluation especially in calcified coronary arteries. OCT provides better spatial resolution and more detailed visualization of calcified lesion and can give more comprehensive information about plaque characteristics including calcium distribution, depth and volume which can be an essential information for procedural planning (17). We've opted for IVUS in this case due to ostial lesion location in both arteries of interest, RCA and LAD, due to possibility of suboptimal image quality if OCT was used due to insufficient blood clearing from the vessel.

In our case, use of combined imaging modalities of CTCA and IVUS provided important information that allowed identification of coronary anomaly type, determination of the extent and distribution of atherosclerotic plaques, as well as optimization of PCI strategy. We considered the result was good after initial PCI procedure. Although we didn't achieve 90% of stent expansion relative to the lumen diameter, we think that further attempts to optimize RCA ostium could increase the risks of RCA ostial dissection and LAD compromise.

PCI of substantially calcified lesions is associated with poor procedural success, greater complication rates, and inferior long-term clinical outcomes (18). The possible options for treatment of very calcified lesions could be rotational or orbital atherectomy, non-compliant and cutting balloons together with IVL. Rotational atherectomy can be challenging in ostial RCA lesions due to several reasons: difficulty of coaxial alignment of guiding catheter against RCA ostium, risk of laceration of the ostium and potential for embolization of rotablation debris (19). We decided to use IVL with the intention to treat calcified lesion in a more focal manner by causing localized fractures of calcified segments of ostial RCA. IVL causes fractures and local dissection even in thicker calcified segments or calcified nodules which allows better lesion preparation and stent expansion (20). Use of IVL prior to stent implantation was also associated with lower rates of major adverse cardiac events (MACE) and stent thrombosis (21). We have reported use of IVL in ostial RCA lesion in a rare coronary anomaly where, in our opinion, use of IVL facilitated the lesion preparation while preventing major ostial dissection that can extend into aorta and/or ostium of LAD. IVUS guidance enabled optimal IVL balloon sizing which led to optimal lithotripsy effect and contributed to procedural success (21). Although, as previously mentioned, IVL is considered safe and potent method of lesion preparation, target-vessel revascularization still occurs in approximately 10% of patients treated with IVL (22). In our case it might be that the deep, concentrated calcium resembling calcified nodule could influence the occurrence of restenosis, despite optimal PC procedure (23). Studies have found that in-stent restenosis (ISR) can be linked to factors like patient characteristics, genetics, stent features, lesion shape, and procedural methods (24). The anatomy of the blood vessels also contributes to restenosis, particularly during PCI procedures involving bifurcation lesions (25). However, there is insufficient data regarding the impact of coronary anomalies, such as the one presented in this case. Using drug-coated balloons (DCB) for the management of in-stent restenosis was linked to lower MACE than treatment with regular, non-coated balloons, although our patient does not resemble study patients treated with DCBs in the studies that evaluated their effectiveness. However, the principles of construction of new generation DCBs seem promising even for complex patients, like ours was (26).

## Conclusion

Although rare, CAAs could be associated with coronary artery disease and usually present additional challenge for interventional treatment. Advanced imaging modalities, including CTCA and IVUS, provide good procedural guidance during complex PCI procedures in patients with CAAs, while IVL provides solutions for calcified lesions, not affected by the variations in coronary anatomy. Lesion restenosis after PCI represents a challenge in everyday clinical practice, and using DCBs for treatment might be a valid option. Whether the unique coronary anatomy in this case played a part in the need for repeated revascularization remains uncertain.

## Data Availability

The raw data supporting the conclusions of this article will be made available by the authors, without undue reservation.
